# Sensory and cognitive functions, gait ability and functionality of older adults*

**DOI:** 10.1590/1518-8345.3499.3282

**Published:** 2020-06-01

**Authors:** Tirso Duran-Badillo, Bertha Cecilia Salazar-González, Juana Edith Cruz-Quevedo, Ernesto Javier Sánchez-Alejo, Gustavo Gutierrez-Sanchez, Perla Lizeth Hernández-Cortés

**Affiliations:** 1 Universidad Autónoma de Tamaulipas (UAMM-UAT), Unidad Académica Multidisciplinaria, Matamoros, Tamaulipas, Mexico.; 2 Universidad Autónoma de Nuevo León, Facultad de Enfermería, Monterrey, Nuevo León, Mexico.; 3 Universidad Veracruzana, Facultad de Enfermería, Veracruz, Veracruz, Mexico.; 4 Universidad Autónoma de Nuevo Léón, Facultad de Agronomia, Escobedo, Nuevo León, Mexico.; 5 Universidad Autónoma de Nuevo León, Facultad de Organización Deportiva, San Nicolás de los Garza, Nuevo León, Mexico.

**Keywords:** Sensation, Gait, Cognition, Activities of Daily Living, Disabled Persons, Aged, Sensação, Marcha, Cognição, Atividades Cotidianas, Pessoas com Deficiência, Idoso, Sensación, Marcha, Cognición, Actividades Cotidianas, Personas con Discapacidad, Anciano

## Abstract

**Objective:**

to know the relationship between the sensory function, gait ability, and cognitive function with dependency in older adults.

**Method:**

a descriptive cross-sectional design, 146 older adults took part.

**Measurements:**

Snellen chart, Audiometer, Stereognosia tests, Semmes-Weinstein monofilament, basic aromas and flavors, GAITRite system, Montreal Cognitive Assessment Test, the Barthel Index, and the Lawton and Brody Index.

**Results:**

sensory function, cognitive function and gait explain 25% dependence on basic activities of daily life and 21% dependence on instrumental activities of daily life. The variables that influence dependence on basic activities were taste (p=.029), gait speed (p=.009), cadence (p=.002) and step length (p=.001) and, in instrumental activities, gait speed (p=.049), cadence (p=.028) and step length (p=.010).

**Conclusion:**

gait speed, cadence and stride length are variables that influence both dependence on basic and instrumental activities of daily life.

## Abstract (pt)

**Objetivo:**

conhecer a relação entre a função sensorial, capacidade de caminhar e função cognitiva com dependência em idosos.

**Método:**

estudo transversal descritivo, com a participação de 146 idosos.

**Medições:**

Carta de Snellen, audiômetro, testes de estereognosia, monofilamento de Semmes-Weinstein, aromas e sabores básicos, sistema GAITRite, *Montreal Cognitive Assessment Test* e índice de Barthel e Lawton e Brody.

**Resultados:**

função sensorial, função cognitiva e caminhar explicam 25% de dependência de atividades básicas da vida diária e 21% de dependência de atividades instrumentais da vida diária. As variáveis que influenciam a dependência de atividades básicas foram: paladar (p=0,029), velocidade de caminhar (p=0,009), cadência (p=0,002) e comprimento do passo (p=0,001), e nas atividades instrumentais, velocidade de caminhar (p=0,049), cadência (p=0,028) e comprimento do passo (p=0,010).

**Conclusão:**

velocidade de caminhar, cadência e comprimento do passo são variáveis que influenciam a dependência de atividades básicas e instrumentais da vida cotidiana.

## Abstract (es)

**Objetivo:**

conocer la relación entre la función sensorial, capacidad de marcha y función cognitiva con la dependencia en adultos mayores.

**Método:**

diseño descriptivo transversal, en que participaron 146 adultos mayores.

**Mediciones:**

Carta Snellen, Audiómetro, pruebas de Estereognosia, Monofilamento de Semmes-Weinstein, aromas y sabores básicos, sistema GAITRite, *Montreal Cognitive Assessment Test* e Índice de Barthel y de Lawton y Brody.

**Resultados:**

la función sensorial, función cognitiva y marcha explican el 25% de la dependencia en las actividades básicas de la vida diaria y 21% de la dependencia en actividades instrumentales de la vida diaria. Las variables que influyen sobre la dependencia en actividades básicas fueron gusto (p=0,029), velocidad de marcha (p=0,009), cadencia (p=0,002) y longitud del paso (p=0,001); y en actividades instrumentales, velocidad de marcha (p=0,049), cadencia (p=0,028) y longitud del paso (p=0,010).

**Conclusión:**

velocidad de marcha, cadencia y longitud del paso son variables que influyen en la dependencia en actividades básicas e instrumentales de la vida diaria.

## Introduction

As age increases, changes arise at the biological level, among which are the decrease in sensory functions, gait ability and cognitive function; these changes become a limitation of the functionality of the older adult that leads them to dependency to carry out basic activities of daily life (BADLs)^(^ 1 ^)^. Some authors report that, in Mexico, between 26.9% and 30.9% of the older adults are dependent^(^ 2 ^-^ 3 ^)^. These data highlight the vulnerability of older adults and the care they require.

The study of dependency in older adults is an important topic, which the nursing professionals should pay attention to, since if it is not properly cared for it can have important individual and family consequences. Among the individual consequences, the quality of life of the older adult stands out and the need for specialized care may arise; at the family level, family dynamics are altered and an increase in physical, emotional and spiritual overload, and in economic expenses^(^ 4 ^-^ 5 ^)^.

The factors associated with dependency are being older, polypharmacy and sedentary lifestyle^(^ 6 ^)^. On the other hand, research studies have been identified that relate vision and hearing problems, as well as taste, smell and touch with dependency^(^ 3 ^,^ 7 ^)^. However, no studies have been identified that jointly evaluate the relationship of each of the senses with this variable. On the other hand, it has been found that there is a relationship between the decrease in gait capacity with dependency, since when there are problems in moving from one place to another, it is not possible to adequately perform the BADLs^(^ 8 ^)^.

Regarding the decrease in the cognitive function, like functional alterations, it has been observed that this is generally manifested by the same aging process^(^ 9 ^)^, or due to the presence of diseases, it has been shown that, at an older age, cognitive problems increase, and these increase if the adult suffers from two or more chronic diseases^(^ 10 ^-^ 11 ^)^; regarding functionality, the literature shows a relationship between cognitive functionality and physical functionality, including gait. Some authors report that poor cognitive performance may be a precursor of the functional limitations that lead to disability^(^ 12 ^-^ 13 ^)^. Memory-related diseases such as dementia or Alzheimer’s, lung disease and arthritis are associated with difficulties in physical functionality and decreased gait speed independently if they occur alone or in combination with other diseases^(^ 11 ^,^ 14 ^)^.

The studies reviewed on risk factors for dependency in older adults address variables such as age, schooling, smoking, and alcoholism; but very few address the sensory function. For this reason, the objective was to know the relationship between sensory function, gait ability, and cognitive function with dependence in older adults. The information obtained will help guide the areas in which nursing actions can be designed to prevent or delay dependency as much as possible.

## Method

A descriptive cross-sectional study was carried out, older adults residing in the urban area, adjacent to a health center belonging to the Health Secretariat of Monterrey, Nuevo León, Mexico, were recruited; the recruitment period was from January to May 2016. The participants were adults 60 years old or older, and the following were considered as inclusion criteria: older adults with the ability to walk, listen and answer the interviewer. The sample consisted of 146 older adults, calculated with the n-Queryadvisor 4.0 statistical package for a correlation coefficient with an effect size of *r*=.14, a power of 90%, and a significance of .05. The sampling was non-probabilistic with the snowball technique.

Visual, auditory, tactile, olfactory and gustatory acuity were assessed for the sensory function. In people who know how to read, visual acuity was measured with the Snellen Chart of letters and, in those who were not, the Snellen chart with drawings was used. The chart was placed at a distance of 6 m from the participant. Normal vision was considered when the parameters were 20/15 or 20/20 and abnormal, when they did not achieve those figures. Hearing acuity was measured with a WelchAllyn 232™ Manual Audiometer. The older adult was asked to indicate when they heard a sound. It was considered normal when the subject indicated listening between -10 dB and 26 dB and with alteration when it was higher. A higher score represents greater hearing impairment.

The acuity of touch was measured through discriminatory sensitivity, the stereognosia test was performed, which measures the person’s ability to identify an object. The participant was put on a mask to prevent him from seeing and later a key was placed in his hand and he was asked what it was. The person had to identify within 5 s, measured with a Steren CLK-150 digital chronometer, if so, it was classified as normal and if it was not identified, as altered. The Semmes-Weinstein monofilament test was used to assess the sensitivity of the back and sole of the feet. With the tip of a 10 g monofilament, one point on the back and nine points on the sole of each foot were touched. The total points perceived by the subject (0 to 10) were considered, where a higher score was rated with greater sensitivity^(^ 15 ^-^ 16 ^)^. This test has shown a Kappa reproducibility of .76 to .96, a sensitivity between 56 and 93.1%, with a specificity of 94.9 and 100%^(^ 17 ^)^.

The olfactory and gustatory acuity was measured with the test of aromas and basic tastes with the Caul selection method, respectively, both proposed by the sensory laboratory of the Agronomy School of the Autonomous University of Nuevo León. For olfactory acuity, 2 g of previously ground ingredients (cumin, pepper, anise, cinnamon and rosemary) were placed in containers; the substances were covered with cotton and the containers were labeled with the corresponding name. The same substances were placed in other containers and the containers were labeled with codes. The subject was instructed to smell from left to right each substance labeled with names and to memorize the aroma, later he was given to smell coffee to neutralize the aromas and was asked to smell those found in the coded containers and say what substance it corresponded to. A summation of the aromas was distinguished, the higher the score was considered the better olfactory acuity.

Taste acuity measures the ability to recognize four basic tastes (sweet, salty, sour, and bitter). Sucrose (16 g/l), sodium chloride (5 g/l), citric acid (1 g/l) and quinine water (.5, undiluted) were used. The substances were weighed on an AND brand analytical scale, HR-200 series, and were subsequently diluted in containers with one liter of water. The diluted substances were placed in 20 ml containers, the containers were coded and presented to each subject to identify the flavor of each one. A bottle of water was given for the subject to rinse or drink as many times as necessary to eliminate the taste from their mouth. A summation was made of the flavors he distinguished, the higher the score, the better the taste acuity was considered. It is worth mentioning that both the basic taste and flavor test with the Caul selection method are tests used for research in the nutritional/feeding field^(^ 18 ^)^; however, their reliability is not reported in the literature.

The characteristics of the gait were measured with the GAITRite system, consisting of an electronic mat 90 centimeters wide and 550 long, connected to a computer equipped with software (Standard GAITRite) in which the footsteps of the older adults were processed. This system reports a reliability for its measurement of 0.91 to 0.99^(^ 19 ^)^. Gait speed, width, step length and cadence were considered.

The Montreal Cognitive Assessment Test (MoCA)^(^ 20 ^)^ was used to measure cognitive decline, which allows examining the cognitive functions. MoCA examines different cognitive abilities through reagents with assigned scores for the criteria to be met in each of them. The reagents and scores are the following: visuospatial/executive level (5 points), identification (3 points), attention (6 points), language (3 points), abstraction (2 points), delayed memory (5 points), and orientation (6 points). The points obtained in each of the skills evaluated must be added together, a score equal to or greater than 26 corresponds to a normal individual, and a lower score classifies it with mild cognitive impairment. The MoCA test has recently been used in the Mexican population^(^ 21 ^)^, was translated into Spanish and reported a Cronbach’s alpha of 0.76^(^ 22 ^)^, the Cronbach’s alpha obtained in this study was .80.

Functionality was measured with the Barthel Index for dependency on basic activities of daily living (BADLs), with a Cronbach’s alpha of .86-.92^(^ 23 ^)^ and with the Lawton and Brody Index for dependency on instrumental activities of daily living (IADLs), with a Cronbach’s alpha of 0.78, and an intraclass reliability of 0.95^(^ 24 ^-^ 25 ^)^. In both instruments, lower scores indicate greater dependency. In this study, Cronbach’s alpha values of .69 and .89 respectively were obtained.

Data was processed and analyzed in the Statistical Package for the Social Sciences (SPSS), version 20 for Windows. Descriptive statistics were used through frequencies and proportions that allowed describing the sociodemographic aspects of the participants, the distribution of the variables with the Kolmogorov-Smirnov goodness-of-fit test with Lilliefors correction, and, based on the results, it was decided to use non-parametric statistics (Spearman’s correlation and Man-Whitney’s U). A general linear model of multivariate contrast was performed where the age, gender, sensory function, gait ability, and cognitive function variables were located as independent variables and dependence on BADLs and IADLs as dependent variables.

The approvals were obtained of the Research and Ethics in Research committee of the Nursing School of the Autonomous University of Nuevo León (Record No.: FAEN-M-1192) and of the committee of the Directorate of Education, Research in Health and Quality of the Ministry of Health of Nuevo León (Record No.: DEISC-19 01 16 01), with the authorization of the Ministry of Health to invite older adults to participate.

## Results

146 older adults were evaluated with a mean age of 68.65 years old (SD=6.69), 78.8% (n=115) women, 58.2% (n=85) with a marital partner, and 32.2% (n=47) that reported having suffered any fall in the last twelve months. 7.5% (n=11) indicated using a gait support device, of which nine used a cane and two, a walker. On average, the older adults reported a schooling of 9.5 years (*SD*=4.96) and indicated taking 2.42 (*SD*=2.21) medications *per* day. Table 1 shows descriptive data.

**Table 1:** Sensory function, gait capacity and functionality. Monterrey, NL, Mexico, 2016

Variable	Mean	SD*	Mdn^†^	95% IC^‡^	
				Lower limit	Upper limit
Age	68.65	6.69	67.50	67.56	69.75
Sensitivity in left foot	9.32	1.74	10.00	9.04	9.61
Sensitivity in right foot	9.27	1.74	10.00	8.99	9.56
Hearing acuity (Left ear)	30.55	11.58	25	28.65	32.44
Hearing acuity (Right ear)	32.19	13.40	30.00	30.00	34.38
Olfactory acuity	2.92	1.64	3.00	2.65	3.19
Taste acuity	2.63	1.23	3.00	2.43	2.83
Gait speed	94.62	20.71	95.80	91.23	98.01
Cadence	104.24	11.14	103.60	102.43	106.07
Step length (Left foot)	53.95	9.27	54.29	52.43	55.47
Step length (Right foot)	54.57	9.19	54.39	53.07	56.07
Step amplitude (Left foot)	9.65	3.05	9.70	9.15	10.15
Step amplitude (Right foot)	9.13	3.01	9.34	8.63	9.62
Cognitive function	21.82	4.83	23.00	21.02	22.61
BADLs^§^	96.16	7.33	100.00	94.96	97.36
IADLs^║^	7.30	1.72	8.00	7.02	7.58

* SD = Standard deviation;

† Mdn = Median;

‡ CI = Confidence interval;

§ ;BADLs = Basic activities of daily life;

║ IADLs = Instrumental activities of daily life

An additional analysis was performed to explore whether there was a significant difference in the variables of sensory function, gait capacity and functionality by age group, less than or equal to 79.9 (n=133) years old and over 80 (n=13), observed a significant difference in auditory, olfactory and gustatory acuity, in addition to gait speed and step length. As expected, adults over 80 are the ones with the greatest alteration. The statistics are shown in Table 2.

**Table 2:** Comparison of means of sensory, gait and functionality variables according to age group. Monterrey, NL, Mexico, 2016

Variable	Mean		*t*	*p*
	< 79.9 (n=133)	≤ 80 (n=13)		
Foot sensitivity	9.39	8.08	1.69	.114
Auditory acuity	31.47	39.62	-3.61	.001
Olfactory acuity	3.12	.85	6.99	.000
Taste acuity	2.66	1.62	2.90	.012
Gait speed	96.52	75.13	3.43	.004
Cadence	104.7	99.00	1.73	.104
Step length	54.82	45.06	3.89	.001
Step amplitude	9.52	10.96	1.86	.082
Cognitive function	22.5	14.77	4.59	.000
BADLs*	96.69	90.38	1.84	.088
IADLs^†^	7.59	5.31	2.61	.022

* BADLs = Basic activities of daily life;

† IADLs = Instrumental activities of daily life

Statistically significant differences were observed in functional dependence. Older adults had greater dependence on BADLs with impaired visual acuity in the test without glasses compared with those without alteration (*U*=456.50; *p*=.031) and older adults with alteration of foot sensitivity compared to those without alteration (*U*=1522.50; *p*=.011). There were no significant differences in visual acuity on the spectacle test, palm sensitivity, and hearing acuity.

Significant differences were found in the IADLs. Older adults presented greater dependence with alteration of the sensitivity of the palm of the hand, compared to those without alteration (*U*=595.00; *p*=.003), those that resulted with impaired foot sensitivity compared to those that did not (*U*=1649.00; *p*=.022) and in those with impaired auditory acuity compared with those without alteration (*U*=2088.00; *p*=.002). No significant differences were observed between visual acuity in the test with and without glasses, gustatory acuity and olfactory acuity.

In Table 3, the correlation coefficient showed a positive association between gait speed and step length with the BADLs, and between gait speed, cadence and step length with the IADLs, and a negative association between step width and the IADLs. A positive association was identified between the cognitive function and the BADLs and IADLs.

**Table 3:** Relationship between gait and cognition characteristics with basic and instrumental activities of daily life. Monterrey, NL, Mexico, 2016

Variable	BADLs*	IADLs^†^
Gait speed (centimeters/second)	.307 (*p* ≤.05)	.365 (*p* ≤.05)
Cadence (steps/second)	.119	.196 (*p* ≤ .01.)
Step length (centimeters)	.346 *(p* ≤.05)	.378 (*p* ≤.05)
Step amplitude (centimeters)	-.101	-.225 (*p* ≤.05)
MoCA^‡^	.236 (*p* ≤.05)	.364 (*p* ≤.05)

* BADLs = Basic activities of daily life,

† IADLs = Instrumental activities of daily life,

‡ MoCA = Montreal Cognitive Assessment test

A linear regression analysis was performed to find out the variables that influence dependency. The independent variables (visual, auditory, olfactory, gustatory and tactile acuity, gait speed, cadence, width and length of the step and cognitive function) explain 25% of the BADLs [*F*(19, 145)=3.578, *p*<.01; R^2^=.25]. The significant variables in this model were taste, gait speed, cadence and step length; a second analysis was performed to determine the variables that influence dependency in the IADLs, the independent variables explain 21% of the dependency [*F*(19, 145)=3.105, *p*<.01; R^2^=.21]. The significant variables in the dependency for the IADLs were gait speed, cadence, and step length (Table 4).

**Table 4:** Influence of personal variables, sensory capacities, gait and cognition on the BADLs (Barthel Index) and on the IADLs (Lawton & Brody Index). Monterrey, NL, Mexico, 2016

Variable	SS*	d^f†^	MS^‡^	F^§^	p^║^
**Barthel: Adjusted R=.253**					
Age	28,762	1	28,762	.721	.397
Sex	17,489	1	17,489	.438	.509
Snellen without glasses	31,775	3	10,592	.265	.850
Audiometry	181,099	3	60,366	1,513	.214
Taste	195,102	1	195,102	4,891	.029
Smell	9,362	1	9,362	.235	.629
Monofilament	272,081	3	90,694	2,273	.083
Stereognosia	53,491	1	53,491	1,341	.249
Gait speed	279,433	1	279,433	7,005	.009
Cadence	384,498	1	384,498	9,638	.002
Length	453,781	1	453,781	11,375	.001
Amplitude	38,904	1	38,904	.975	.325
MoCA^¶^	57,190	1	57,190	1,434	.233
**Lawton and Brody: Adjusted R=.216**					
Age	5,566	1	5,566	2,379	.125
Gender	5,432	1	5,432	2,322	.130
Snellen without glasses	3,315	3	1,105	.472	.702
Audiometry	4,614	3	1,538	.657	.580
Taste	7,297	1	7,297	3,119	.080
Smell	.703	1	.703	.301	.585
Monofilament	.661	3	.220	.094	.963
Stereognosia	8,214	1	8,214	3,511	.063
Gait speed	9,250	1	9,250	3,954	.049
Cadence	11,501	1	11,501	4,916	.028
Length	16,224	1	16,224	6,935	.010
Amplitude	4,648	1	4,648	1,987	.161
MoCA^¶^	1,226	1	1,226	.524	.470

* SS = Sum of squares;

† df = Degrees of freedom;

‡ MS = Mean square;

§ F = Fisher's F distribution;

║ *p* = Significance;

¶ MoCA = Montreal Cognitive Assessment test

## Discussion

In the present study it was found that the dependence for the BADLs is different in older adults with and without alteration of visual acuity, a finding which is consistent with an article on a Mexican population^(^ 3 ^)^. This is consistent in populations of foreign countries; in Tokyo and Georgia, a study was carried out to find out the predictors of dependency and it was found that vision predicts in up to 60% the alteration in functionality^(^ 26 ^)^, this is why the evaluation of visual acuity must be present in the medical evaluations that are carried out on older adults to correct the alteration as much as possible and avoid dependencies due to this cause. Likewise, it was observed that the dependence for the BADLs and the IADLs is different in the subjects with and without alteration of the tactile acuity in the feet, this takes coherence since both activities include performing displacement actions (gait, climbing stairs) which are affected when somatic sensory changes occur^(^ 7 ^)^. It would be worth assessing these variables in adults with pathologies that alter the CNS, such as diabetes, for example.

In the search for differences regarding the dependence to perform the BADLs in older adults with and without alteration of auditory acuity, no statistically significant differences were found^(^ 26 ^)^. This may be because, for the activities of daily life, they focus on mobility that needs coordination and proprioception, which requires vision and balance and not so much the auditory system, another explanation may be that adults acquire strategies to adapt to the environment and perform their activities daily. However, it was identified that dependence on the IADLs is different in older adults with and without alteration of auditory acuity, coincides with the authors who evaluated the relationship of vestibular sensitivity with ADLs, this may be due to the fact that people with hearing loss show greater difficulty in generating internal representations of the outside world^(^ 27 ^)^; in this sense, the IADLs require communication with other people, such as using the phone, transportation, finance and shopping.

Likewise, a positive association was found in the BADLs with gait speed, step length and in the IADLs with gait speed, cadence and step length. A negative relationship was observed between the amplitude of the step and the IADLs. These data coincide with studies carried out at the international level, where it is indicated that the ability to walk is related to the dependence to perform the ADLs^(^ 7 ^,^ 28 ^-^ 29 ^)^. In this sense, it has been reported that older adults with slow gait speed have greater difficulty in performing the ADLs^(^ 8 ^)^. This happens because, as a consequence of aging, there are changes in the systems, such as the nervous, skeletal, visual, vestibular and proprioceptive systems, which intervene with postural control and gait, which causes alteration of functionality and leads to dependency^(^ 30 ^)^.

Another finding was that the lower the cognitive function, the greater the dependency to perform the BADLs and the IADLs. The above explains that, when mild cognitive impairment occurs, in older adults independence is altered to carry out daily activities, this is due to the alteration in simple and complex cognitive processes that prevents the interpretation of stimuli to turn them into a response^(^ 31 ^-^ 32 ^)^.

Older adults with normal visual acuity in the test with and without glasses, presented greater cognitive function. Likewise, older adults with normal gustatory and auditory acuity had a better cognitive function. The significance found between auditory acuity and cognitive function agrees with that reported by other authors^(^ 33 ^-^ 37 ^)^. The findings of the present study confirm what is established in the literature, where it is indicated that there is a relationship between taste and smell with cognitive function^(^ 38 ^)^.

Older adults with normal visual acuity in the test without glasses, presented higher gait speed and step length, compared to those who presented visual acuity with alteration, gait alterations often presented by visual and vestibular deficits^(^ 39 ^)^. Older adults with normal tactile acuity had better step length and width, and those with normal hearing acuity had better gait speed and step length. This relationship arises because the main components of gait are balance and locomotion, which depend on vestibular and somatic sensory input, that is, sight, hearing and touch^(^ 5 ^,^ 28 ^-^ 29 ^)^.

Older adults with higher gait speed, longer length and shorter gait width resulted in greater cognitive function, these findings are consistent with that reported by other authors who show statistically significant association between gait speed and stride length with executive^(^ 40 ^)^; likewise, in a review of the literature they report that there is an association between gait and cognition in older adults^(^ 41 ^)^. Along these same lines, the literature shows that proprioceptive input is required to execute the gait process ^(^ 7 ^,^ 28 ^-^ 29 ^)^, where the brain receives information on the position and movement of the different parts of the body involved in the gait. Therefore, the above explains that the gait parameters are altered when deterioration of simple and complex cognitive processes occurs.

Finally, multivariate regression analysis showed that taste, gait speed, cadence, and step length affect the performance of the BADLs, and speed, cadence, and step length affect dependence on performing the IADLs. In the Roper, Logan and Tierney Nursing Model^(^ 42 ^)^ it is stated that there are different factors that influence dependency, among which is the biological factor that includes aging, sensory function, gait ability and cognitive function, which is supported by the findings in the present, therefore, it is considered necessary that in the evaluation of the elderly the sensory function, gait and cognition are considered. The study provides evidence on sensory functions such as sight, hearing and touch in the elderly population and their relationship with functional capacities such as gait, activities of daily living and cognition. It is recognized that having used non-probability sampling is a limitation for the generalization of the results; however, what was found in this study is considered an important contribution for both research and the nursing practice, since this information expands the knowledge of the dependency phenomenon and allows to guide or modify the nursing interventions focused on the prevention of dependency in the basic and instrumental activities of daily life in older adults. It is suggested to continue with the study of the function in each of the senses and the affectations of these by age group and pathologies that are suffered. It has been documented that dependency can be related to depression, social environment, physical environment of the home, and usability in the home, falls and body weight, so it is recommended to carry out another study that addresses these variables. On the other hand, it would be interesting to explore the adaptations and strategies that older adults adopt to carry out the activities of daily life despite the limitations.

## Conclusion

Taste, gait speed, cadence, and step length were found to affect independence/dependency for performing the BADLs; and speed, cadence, and step length affect dependency for performing the IADLs. Therefore, older adults with greater affectation have a higher dependency risk.

## References

[1] Soria-Romero Z, Montoya-Arce BJ (2017). Aging and factors associated with quality of life for elderly
people in State of Mexico. Pap Poblac.

[2] Gutiérrez JP, Rivera-Dommarco J, Shamah-Levy T, Villalpando-Hernández S, Franco A, Cuevas-Nasu L (2012). Encuesta Nacional de Salud y Nutrición 2012. Resultados
Nacionales.

[3] Manrique-Espinoza B, Salinas-Rodríguez A, Moreno-Tamayo K, Téllez-Rojo MM (2011). Functional dependency and falls in elderly living in poverty in
Mexico. Salud Publica Méx.

[4] Galvis-López CR, Aponte-Garzón LH, Pinzon-Rocha ML (2016). Perception of the quality of life of caregivers of patients
attending a program for the chronically, Villavicencio,
Colombia. Aquichan.

[5] Salazar-Barajas ME, Garza-Sarmiento EG, García-Rodríguez SN, Juárez-Vázquez PY, Herrerra-Herrera JL, Duran-Badillo T (2019). Funcionamiento familiar, sobrecarga y calidad de vida del
cuidador del adulto mayor con dependencia funcional. Enferm Univ.

[6] Cano-Gutiérrez C, Bordaz Miguel G, Reyes-Ortiz C, Arciniegas AJ, Samper-Ternent R (2017). Assessment of factors associated with functional status in 60
years-old and older adults in Bogotá, Colombia. Biomédica.

[7] Gutierres-Robledo LM, García-Peña C, Medina-Campos R, Parra-Rodriguez L, Lopez-Ortega M, Gonzalez-Meljem JM (2018). Estudio de carga de la enfermedad en personas adultas mayores: un reto
para México. [Internet]..

[8] Durán-Badillo T, Hernández Cortés PL, Guevara-Valtier MC, Gutierrez-Sánchez G, Martínez-Aguilar ML, Salazar-Barajas ME (2019). Gait capacity and functional dependency among older adults with
visión disturbances. Enferm Univ.

[9] Díaz-Venegas C, Wong R (2016). Trajectories of limitations in activities of daily living among
older adults in Mexico, 2001-2012. Disabil Health J.

[10] Segura-Cardona A, Cardona-Arango D, Segura-Cardona A, Muñoz-Rodriguez D, Jaramillo-Arroyave D, Lizcano-Cardona D (2018). Factors associated with the cognitive vulnerability of older
adults in three Colombian cities. Aquichan.

[11] Stenholm S, Westerlund H, Head J, Hyde M, Kawachi I, Pentti J (2015). Comorbidity and functional trajectories from midlife to old age:
the health and retirement study. J Gerontol A Biol Sci Med Sci.

[12] Connolly D, Garvey J, McKee G (2017). Factors associated with ADL/IADL disability in community dwelling
older adults in the Irish longitudinal study on ageing
(TILDA). Disabil Rehabil.

[13] Paredes-Arturo YV, Yarce Pinzon E, Aguirre Acevedo DC (2018). Functionality and associated factors in the older adult of the
city of San Juan de Pasto, Colombia. Rev Cienc Salud.

[14] Flavey JR, Gustavson AM, Price L, Papazian L, Stevens-Lapsley JE (2019). Dementia, comorbidity, and physical function in the Program
All-Inclusive Care for the Elderly. J Geriatri Phys Ther.

[15] Calderón-Campos KM, Parodi JF, Runzer-Colmenares F (2019). Neurological comorbidities and its association with gait speed in
older adults of the Naval Medical Center Cirujano Mayor Santiago Távara
2010-2015. Rev Neuropsiquiatr.

[16] Mendoza-Romo MA, Ramírez-Arriola MC, Velasco-Chávez JF, Nieva-de Jesús RN, Rodríguez-Pérez CV, Valdez-Jiménez LA (2013). Sensitivity and specificity of a utility model of the detection
of diabetic neuropathy. Rev Med Inst Mex Seguro Soc.

[17] Delgado-Díaz DC, Herrera E, Camargo D (2004). La prueba de los monofilamentos: una alternativa para la
detección oportuna del riesgo de pie diabético. Rev Salud UIS.

[18] Duarte C, Ortega A, Trujillo L, Oliva A (2008). Metodología para la formación de comisiones de evaluación
sensorial en café. Cien Tecnol Alim.

[19] Webster K, Wittwer J, Feller J (2004). Validity of the GAITRite walkway system for the measurement of
averaged and individual step parameters of gait. Gait Posture.

[20] Loureiro C, García C, Adana L, Yacelaga T, Rodriguez-Lorensana A, Maruta C (2018). Use of the Montreal Cognitive Assessment (MoCA) in Latin America:
a systematic review. Rev Neurol.

[21] Leiva-Caro JA, Salazar-González BC, Gallegos-Cabriales EC, Gómez-Méza MV, Hunter KF (2015). Connection between competence, usability, environment and risk of
falls in elderly adults. Rev. Latino-Am Enfermagem.

[22] Delgado C, Araneda A, Behrens MI (2019). Validation of the Spanish-language version of the Montreal
Cognitive Assessment test in adults older than 60 years. Neurología.

[23] Cid-Ruzafa J, Damián-Moreno J (1997). Evaluating physical incapacity: the Barthel Index. Rev Esp Salud Pública.

[24] Mendoza-Parra S, Merino JM, Barriga OA (2009). Identificación de factores de predicción del incumplimiento
terapéutico en adultos mayores hipertensos de una comunidad del sur de
Chile. Rev Panam Salud Pública.

[25] Trigás-Ferrín M, Ferreira-González L, Meijide-Míguez H (2011). Scales for the functional assessment in the
elderly. Galicia Clin.

[26] Martin P, Gondo Y, Arai Y, Ishioka Y, Woodard JL, Poon LW (2018). Physical, sensory, and cognitive functioning among centenarians:
a comparison between the Tokyo and Georgia centenarian
studies. Qual Life Res.

[27] Semenov YR, Bigelow RT, Xue QL, Lac SD, Agrawal Y (2015). Association between vestibular and cognitive function in US
adults: data from the National Health and Nutrition Examination
Survey. J Gerontol A Biol Sci Med Sci.

[28] Kerrigan DC, Todd MK, Della Croce U, Lipsitz LA, Collins JJ (1998). Biomechanical gait alterations independent of speed in the
healthy elderly: evidence for specific limiting impairments. Arch Phys Med Rehabil.

[29] Rodriguez-G, Burga-Cisneros D, Cipriano G, Ortiz PJ, Tello T, Casas P (2017). Factors associated with slow walking speed in older adults of a
district in Lima, Peru. Rev Peru Med Exp Salud Publica.

[30] Silva-Fhon JR, Partezani-Rodrigues R, Miyamura K, Fuentes-Neira W (2019). Causes and factors associated to falls among the
elder. Enferm Univ.

[31] Castro-Suarez S (2018). Healthy aging and cognitive impairment. Rev Neuropsiquiatr.

[32] Shimada H, Makizako H, Lee S, Doi T, Lee S, Tsutsumimoto K (2016). Impact of cognitive fraility on daily activities in older
persons. J Nutr Health Aging.

[33] Dupuis K, Pichora-Fuller MK, Chasteen AL, Marchuk V, Singh G, Smith SL (2015). Effects of hearing and vision impairments on the Montreal
Cognitive Assessment. Neuropsychol Dev Cogn B Aging Neuropsychol Cogn.

[34] Mitoku K, Masaki N, Ogata Y, Okamoto K (2016). Vision and hearing impairments, cognitive impairment and
mortality among long-term care recipients: a population-based cohort
study. BMC Geriatr.

[35] Maharani A, Dawes P, Nazaroo J, Tampubolon G, Pendleton N, Sense-Cog WP1 Group (2019). Visual and hearing impairments are associated with cognitive
decline in older people. Age Ageing.

[36] Davidson JGS, Guthrie DM (2019). Older adults with a combination of vision and hearing impairment
experience higher rates of cognitive impairment functional dependence, and
worse outcomes across a set of quality indicators. J Aging Health.

[37] Valero-García J, Casaprima V, Dotto G, Ithurralde C, Lizarraga A, Ruiz V (2015). Relationship between hearing and cognition during aging: a study
of a geriatric population of Rosario. Rev Fed Argent Soc Otorrinolaringol.

[38] Churnin I, Qazi J, Fermin CR, Wilson JH, Payne SC, Mattos JL (2019). Association between olfactory and gustatory dysfunction and
cognition in older adults. Am J Rhinol Allergy.

[39] Jahn K, Zwergal A, Schniepp R (2010). Gait disturbances in old age: classification, diagnosis, and
treatment from a neurological perspective. Dtsch Arztebl Int.

[40] Holtzer R, Wang C, Verghese J (2012). The relationship between attention and gait in aging: facts and
fallacies. Motor Control.

[41] Ambrose AF, Noone ML, Pradeep VG, Johnson B, Salam KA, Verghese J (2010). Gait and cognition in older adults: insights from the Bronx and
Kerala. Ann Indian Acad Neurol.

[42] Roper N, Logan W, Tierney AJ (2000). The Roper-Logan-Tierney model of nursing: based on activities of
living.

